# Overexpression of *TaCR4-A* positively regulates grain size in *Triticum aestivum*

**DOI:** 10.1186/s12870-025-07345-5

**Published:** 2025-10-01

**Authors:** Qian Qian, Weiguo Hu, Yang Yu, Yang Liu, Yesong Tian, Xiaohong Zhang, Donghong Min

**Affiliations:** 1 State Key Laboratory of Crop Stress Biology for Arid Areas, College of Agronomy, Northwest A&F University, Yangling, Shaanxi 712100 China; 2 Institute of Wheat, Henan Academy of Agricultural Sciences, Zhengzhou, Henan 450002 China

**Keywords:** *TaCR4-A*, CRINKLY4 receptor-like kinase, Grain size, *Triticum aestivum*

## Abstract

**Background:**

Grain size is a critical factor that affects yield in wheat (*Triticum aestivum*). CRINKLY4 (CR4) receptor-like kinase regulates epidermal cell differentiation and seed development in several plant species, yet its functional role in wheat remains to be clarified.

**Results:**

In this study, we identified the *TaCR4* gene, which encodes the CRINKLY4 receptor-like kinase homolog in wheat. We created overexpressing (OE) wheat lines with increased *TaCR4-A* expression and found that the grain length, width, thickness, and weight of the OE lines increased. Cytological analysis revealed that the overexpression of *TaCR4-A* increased the length, width, and area of wheat grain outer pericarp cells.

**Conclusions:**

This study demonstrated that *TaCR4* positively regulates wheat grain size by regulating the size of grain epidermal cells.

**Supplementary Information:**

The online version contains supplementary material available at 10.1186/s12870-025-07345-5.

## Background

Wheat (*Triticum aestivum* L.) is one of the most important staple crops worldwide. Yield is pivotal to global food security, and grain size and weight are important factors in crop productivity. Increasing yield is a key goal in wheat breeding, and understanding the molecular mechanisms that genetically regulate wheat grain size is crucial. Several genes that regulate grain size have been identified in wheat, such as *TaMYB44* [1], *Fructokinase* (*TaFRK2-7B1*) [2], *Cytokinin Oxidase/Dehydrogenase* (*TaCKX11-D*) [3], *Gibberellin 3-Oxidase* (*GA3OX*) [4], *Flowering Locus T* (*TaFT-D1*) [5], *TaWUS-like-5D* [6], *Growth-Regulating Factor 4* (*TaGRF4*) [7], *TaGS3* [8], *Enhancer Of GS2AA* (*TaEOG1*) [9], *TaDA1* [10], *Grain Size 5 − 3* (*TaGS5-3 A*) [11], *Grain Width and Weight 2* (*TaGW2-A1* and *TaGW2-6 A*) [12, 13], *Cytokinin Oxidase/Dehydrogenase 6* (*TaCKX6-D1*) [14] (https://graingenes.org/GG3/wgc), etc. Grain size is determined by grain length, width, and thickness, and its genetic regulatory mechanism needs to be further elucidated.

Plant receptor-like kinases (RLKs) are transmembrane proteins with extracellular domains, a single transmembrane region and intracellular kinase domains [15, 16]. RLKs play a crucial roles in many aspects of plant growth and development, participating in the regulation of cell division and expansion, and some RLKs affect the size of plant seeds [17, 18]. These include mini seed 2 (MIS2, OsCR4), the putative Brassinosteroid-Signalling Kinase (OsBSK2) and S-domain receptor-like protein kinase (OsGRSK1) in rice [19–21]; pod size/weight1 (PSW1) in peanuts [22]; receptor-like kinase (ZmRLK7) in maize [23]; somatic embryogenesis receptor kinases (TaSERKs) in wheat [24]; lectin receptor-like kinase (LecRK)-VIII.2 and ERECTA (ER) in Arabidopsis [25, 26]. However, the exact mechanism by which RLKs control seed size remains to be further studied.

*Crinkly4* (*CR4*) belongs to the *receptor-like kinase* (RLK) gene family and encodes a receptor-like kinase that regulates cell differentiation in epidermis-related tissues [19, 27, 28]. *Crinkly4* was first identified in maize, with *cr4* mutants producing wrinkled leaves and aleurone-deficient grains [27]. *Arabidopsis Crinkly4* (*ACR4*) mutations cause various defects in epidermal-related cells, such as seed coats and leaf epidermis [28, 29]. Overexpression of antisense *ACR4* cDNA resulted in a loss of function and varied degrees of embryonic malformation [30]. In rice, knocking down *OsCR4* using RNA interference caused the palea and the lemma to separate at later spikelet stages, damaged embryo and endosperm development, and led to aleurone defects [31]. The *mini seed2* (*mis2*,* OsCR4*) mutant has reduced grain length, width, thickness, and a wrinkled surface. *OsCR4* controls the grain size by coordinating and regulating the size and number of epidermal cells. After the complete *OsCR4* genomic DNA was transferred into the mutant, its large-grained, smooth-surfaced, closed-shell phenotype was completely restored [19]. Research on *Crinkly4* in wheat is limited and requires further investigation.

This study identified the *TaCR4-A* gene in wheat, which encodes the CRINKLY4 homolog. Following its overexpression of *TaCR4-A* in wheat, the grain length, width, thickness and weight all increased. Cytological analysis revealed that the epidermal cells of grains in overexpressing lines were enlarged, with no significant change in cell number. These results indicate that TaCR4-A positively regulates wheat grain size by regulating epidermal cell size.

## Methods

### Plant materials and growth conditions

Wheat (*Triticum aestivum* L.) cultivar Kenong 199 (KN199) was used for gene isolation, gene expression analysis and gene transformation. The wheat used in this study was planted on October 19, 2024, in the experimental field of Northwest A&F University, Shaanxi Province. Each wheat Line comprised three rows, with each row being a length of 2 m, a row spacing of 25 cm, and a plant spacing of 5 cm. A randomized block design with three replicates was employed. Field Management followed local practices. For generation advancement, wheat was grown in greenhouses under a 16 h light/8 h dark cycle at 20/22 ℃. Tissues were frozen in liquid nitrogen immediately after collection and stored at −80 °C until RNA isolation.

### Gene identification and phylogenetic analysis

The *TaCR4* coding sequences were obtained from the wheat whole genome sequencing database in Ensemble Plants (http://plants.ensembl.org/) [32]. Sequence alignment and analysis were performed using DNAMAN (https://www.lynnon.com/). Protein domain prediction was performed using the SMART online tool (http://smart.embl.de/) [33]. Phylogenetic analysis was conducted using the neighbour-joining method with 1000 bootstrap replicates in MEGA11 [34]. All protein sequences are provided in Supplementary Data S1.

### Total RNA isolation and quantitative real-time PCR (qPCR) analysis

Total RNA was extracted from wheat grain tissues using RNAiso Plus (TaKaRa, Japan). The quality and concentration of the total RNA were assessed using a 1% agarose gel electrophoresis and a NanoDrop 2000 spectrophotometer (Thermo Fisher Scientific, USA). First-strand cDNA was synthesized using the PrimeScript RT Reagent Kit (TaKaRa, Japan). Quantitative real-time PCR (qPCR) was performed using a QuantStudio 3 Flex Real-Time PCR System (Life Technologies, USA) with TB Green Premix Ex Taq II (TaKaRa, Japan). Each reaction included three biological replicates from independent plants per line and three technical replicates. *TaActin* served as the internal reference gene. Relative gene expression levels were calculated via the 2^−ΔΔCt^ method [35]. All primer sequences are provided in Supplementary Table S1.

### Wheat transformation

The *TaCR4-A* coding sequence (CDS) was amplified from wheat grain cDNA and ligated into the pWMB003 vector to generate the Ubi: *TaCR4* overexpression construct. These recombinant plasmid constructs were then transformed into KN199 embryogenic calli via particle bombardment, according to the method described by Xiao et al. [36]. The positively transformed plants were identified using PCR and qRT-PCR. The primer sequences are provided in Supplementary Table S1. The T3 generation of transgenic wheat was used for all subsequent experiments.

### Grain phenotype analysis

To determine the size of the wheat grains, Mature grains were air-dried at 40 °C for 36 h and then stored at room temperature until analysis. The length, width and thickness of each grain were measured using a Vernier caliper with an accuracy of 0.02 mm, and the thousand-grain weight (TGW) was measured using an electronic analytical balance with an accuracy of 0.0001 g. Thirty fully mature grains were randomly sampled from at least five individual plants of the KN199 and *TaCR4-A* overexpression (OE) lines for measurement.

### Cytological characterization analysis

To observe and measure the size of pericarp cells in wheat grains, we collected grains from the KN199 and *TaCR4-A* OE Lines at 20 days after pollination (DAP). Samples were immediately frozen in Liquid nitrogen and then sliced transversely using a frozen slicer. The middle sections of the sliced samples were stained with a 0.1% (w/v) solution of Fluorescent Brightener 28 for 30 min [37]. Fluorescence images were acquired using a fluorescence microscope (Zeiss, Germany) and stitched together using the MosaicJ plugin in ImageJ [38]. The length, width, and area of the outer pericarp cells were measured using ImageJ, with at least 100 outer pericarp cells were measured from 20 grains collected from five independent plants.

### Statistical analysis

Statistical analysis was performed using the GraphPad Prism 9.0.0 (GraphPad Software, USA, https://www.graphpad.com/). One-way ANOVA and Tukey’s multiple comparison tests were used to calculate the significance. All data are presented as mean ± SD, and different lowercase letters indicate statistically significant differences (*p* < 0.05). At least three biological replicates were analyzed for each measurement.

## Results

### Identification of TaCR4 in hexaploid wheat

**Fig. 1:**
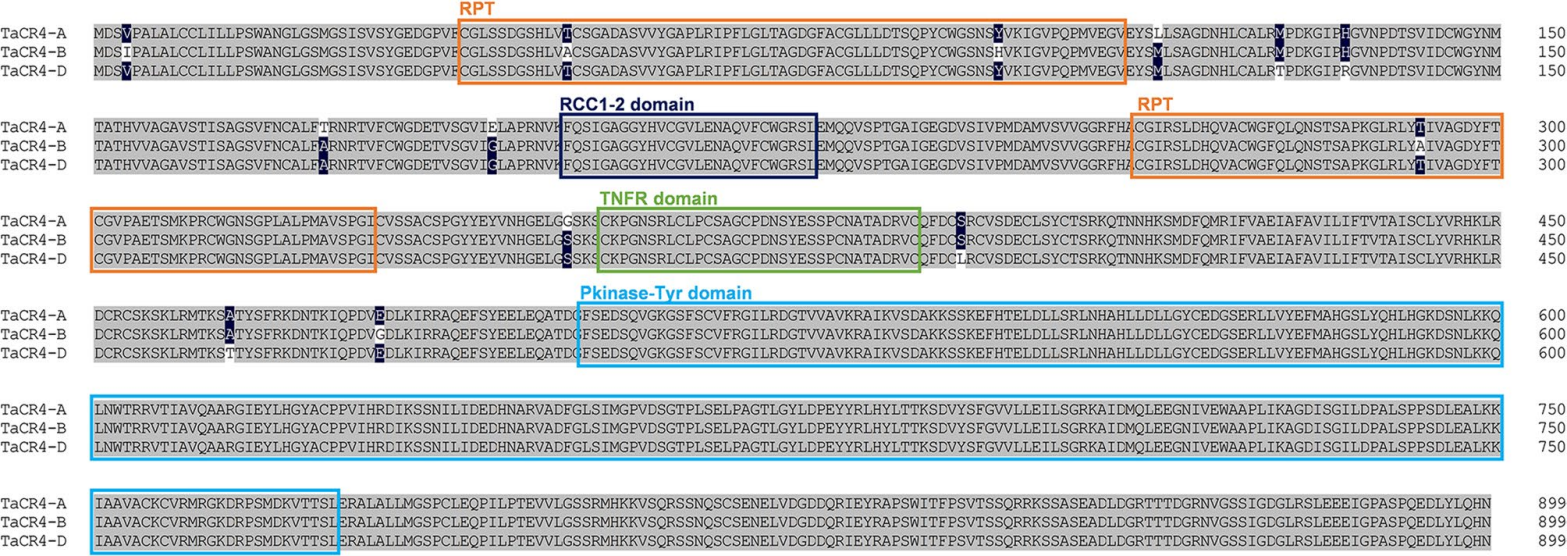
Multiple alignment of the amino acid sequences of TaCR4-A, TaCR4-B and TaCR4-D. The orange box represents the RPT (internal repeat), the dark blue box represents the RCC1_2 (regulator of chromosome condensation repeat) domain, the green box represents the TNFR (tumor necrosis factor receptor) domain and the light blue box represents the Pkinase_Tyr (protein tyrosine and serine/threonine kinase) domain

**Fig. 2:**
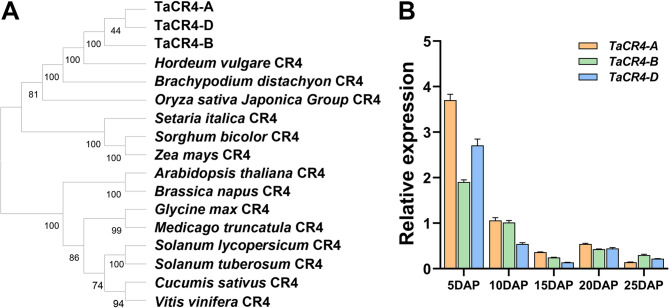
Identification of *Triticum aestivum* TaCR4 homologs and *TaCR4* expression patterns during grain filling stages. **A** Phylogenetic analysis of three TaCR4 proteins and CR4 homologs from other species. **B** Expression patterns of *TaCR4-A*, *TaCR4-B* and *TaCR4-D* at different grain filling stages. Data are means ± SD (*n* = 3); different letters indicate significant differences (*P* < 0.05, one-way ANOVA, Tukey’s test). DAP: days after pollination

The *Crinkly4* gene encodes a receptor kinase that has been identified in rice and maize as being associated with grain size. Based on feature domains and protein sequences, we identified three homologous proteins in wheat, encoded by the genes *TaCR4-A*, *TaCR4-B*, and *TaCR4-D* (TraesCS7A02G301300, TraesCS7B02G201600, TraesCS7D02G296700), which are located on chromosomes 7 A, 7B, and 7D, respectively. A comparison of the amino acid sequences of TaCR4-A/B/D showed 99.52% similarity. The characteristic structures include an intracellular Pkinase_Tyr (protein tyrosine and serine/threonine kinase) domain, and the extracellular RCC1_2 (regulator of chromosome condensation repeat) domain, a TNFR (tumor necrosis factor receptor) domain and two RPTs (internal repeats) (Fig. 1). The amino acid sequences of the kinase_Tyr domain, RCC1_2 domain and TNFR domain in TaCR4-A/B/D were completely consistent. The two RPT domains were identical between TaCR4-A and TaCR4-D, whereas the sequence identities for the respective RPT domains between TaCR4-A and TaCR4-B were 97.18% and 98.55%. This study performed a phylogenetic analysis using TaCR4 and CR4 homologs from other species to analyze their evolutionary relationships. The results revealed that TaCR4 is closely related to HvCR4 in barley, BdCR4 in *Brachypodium distachyon* and OsCR4 in rice (Fig. 2A). To analyze the expression pattern of *TaCR4* during wheat grain filling, we quantified the expression levels of *TaCR4-A*, *TaCR4-B* and *TaCR4-D* in wheat grains at 5, 10, 15, 20 and 25 DAP using RT-PCR. The results showed that *TaCR4* was continuously expressed throughout the wheat filling period, with its expression level decreasing over time (Fig. 2B). Compared with *TaCR4-B* and *TaCR4-D*, *TaCR4-A* presented the highest expression level in grains, and its protein sequence was more homologous to OsCR4. Therefore, we cloned the *TaCR4-A* gene from hexaploid wheat for subsequent analysis.

### TaCR4-A positively regulates grain size

**Fig. 3:**
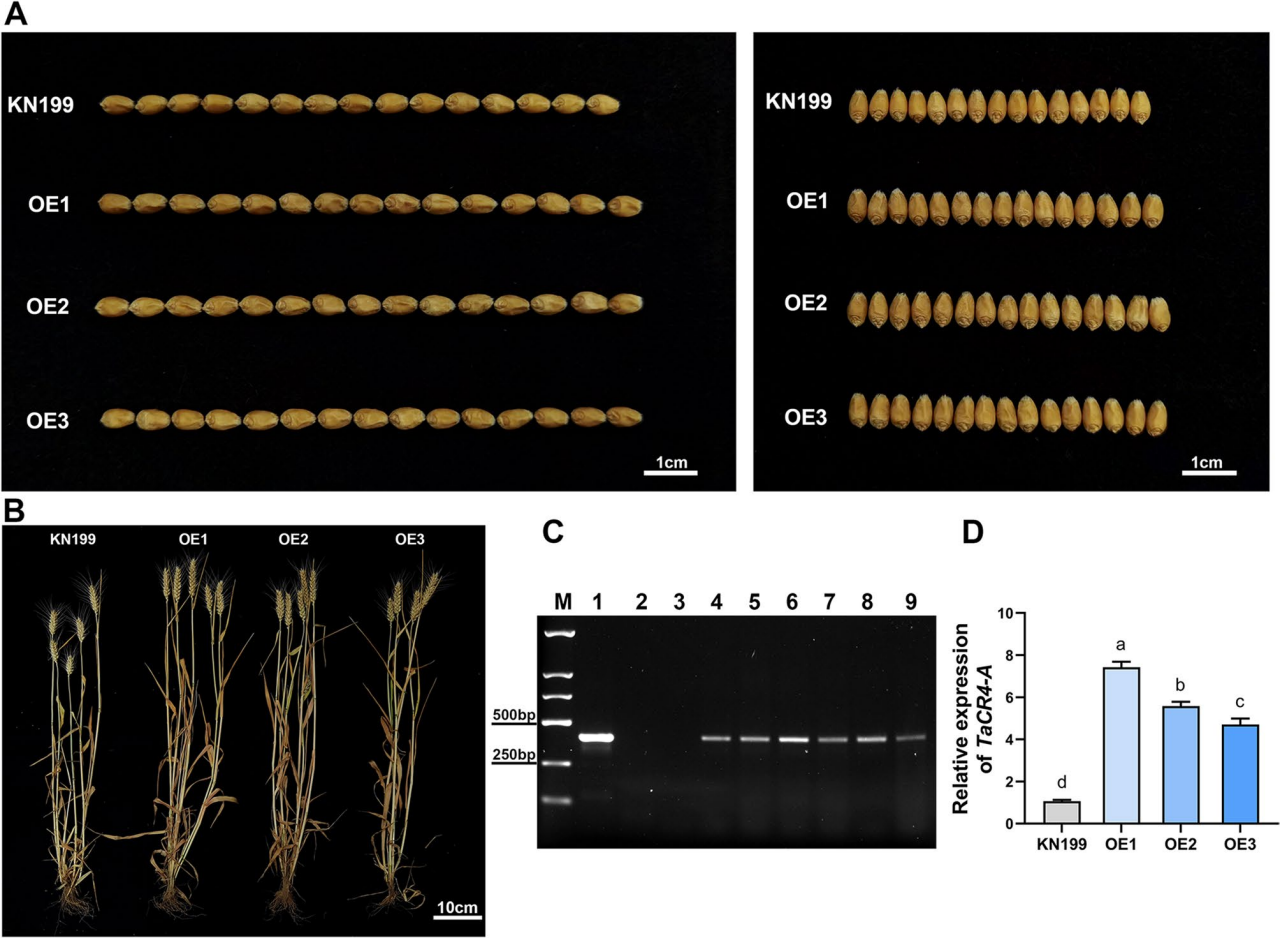
Grain phenotypes of *TaCR4-A* overexpression lines in *Triticum aestivum. ***A** Grain phenotypes of KN199 and *TaCR4-A* OE lines in wheat. Grains were collected from the middle of the spike. **B** Mature plants of KN199 and *TaCR4-A* OE lines. **C** Molecular identification of transgenic wheat by PCR. M: DNA marker DL2000; 1: Ubi: *TaCR4-A* plasmids; 2: H_2_O (negative control); 3: KN199 (wild type); 4–9: different *TaCR4-A OE* lines. **D** Relative expression levels of the *TaCR4-A* OE lines were evaluated by RT-qPCR (*n* = 3). Grain samples were collected at 5DAP. Data are means ± SD (*n* = 3), different letters indicate statistically significant differences, (*P* < 0.05, one-way ANOVA, Tukey’s test)

**Fig. 4:**
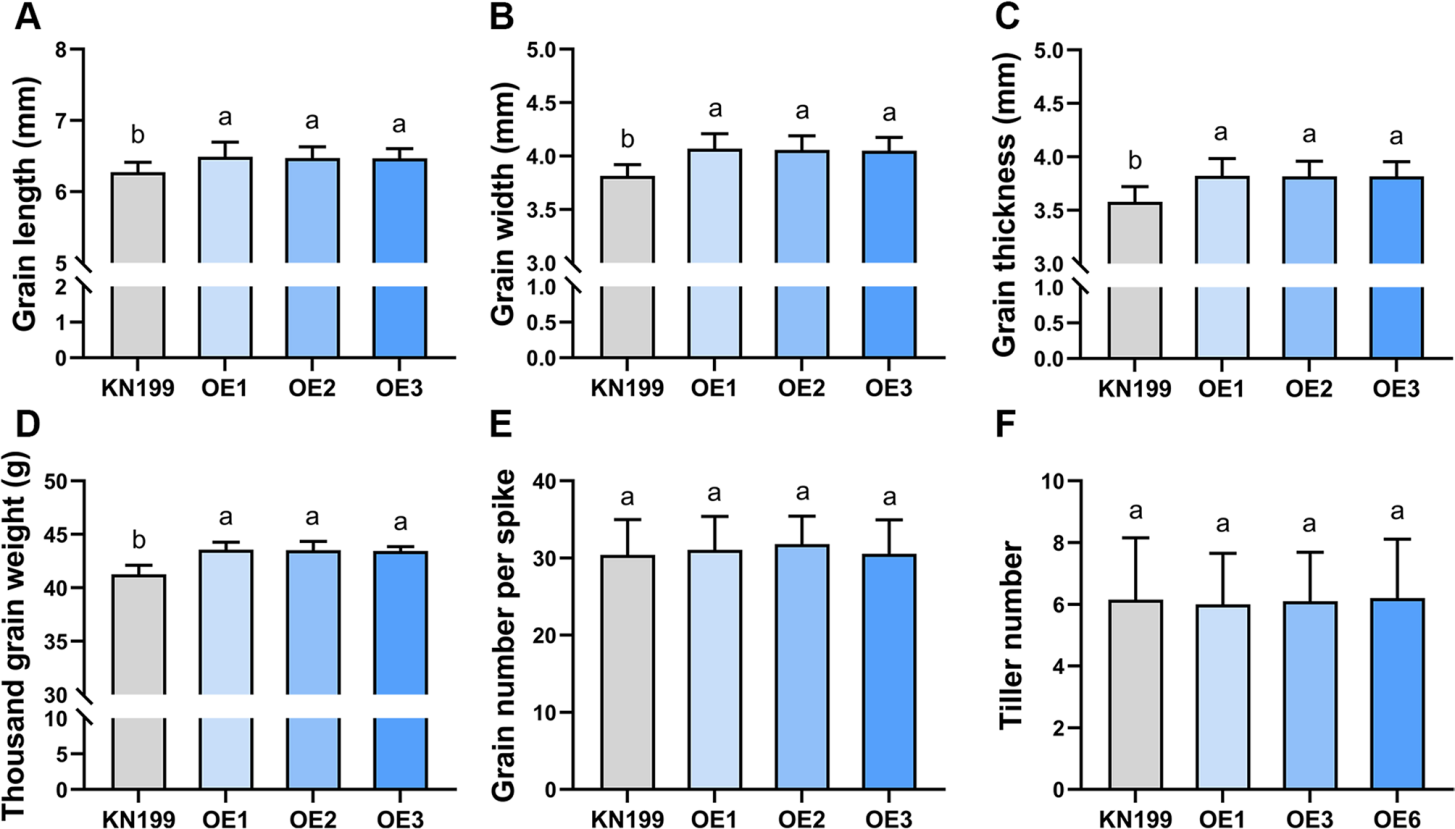
Measurement of grain size and agronomic traits of KN199 and *TaCR4-A* OE lines. **A**-**C** Measurements of grain length (**A**), grain width (**B**) and grain thickness (**C**). Grains (*n* = 30) for measurement were taken from 5 independent plants per line. **D** Measurement of thousand grain weight. Grains were collected from at least 30 independent plants per line. **E**-**F** Measurement of plant height (**E**) and grain number per spike (**F**) (*n* = 20 plants). Data (**A**) to (**F**) are means ± SD, different letters indicate statistically significant differences (*P* < 0.05, one-way ANOVA, Tukey’s test)

To verify the role of *TaCR4* in regulating wheat grain size, we generated *TaCR4-A* overexpressing wheat lines. The Ubi: *TaCR4-A* overexpression vector was constructed, and the plasmid was transformed into the hexaploid wheat variety KN199 using the particle bombardment method. The PCR and qRT-PCR results revealed that the transformation had been successful and that the expression level had increased (Fig. 3C and D). Three wheat OE lines with high expression levels (OE1, OE2 and OE3) were selected for further verification of the role of *TaCR4-A* in regulating wheat grain size. The overexpression lines and KN199 were cultivated under the same conditions and fully mature grains were collected for phenotypic identification. The results showed that wheat grain size increased significantly with elevated *TaCR4-A* expression (Figs. 3A and B). Compared with KN199, *TaCR4-A* OE Lines increased grain length by 3.09–3.48%, grain width by 6.21–6.69%, grain thickness by 6.24–6.76%, and thousand kernel weight by 5.28–5.57% (Figs. 4A-D; Table 1). Moreover, we measured several other agronomic traits simultaneously. Apart from increased plant height and spike length in OE lines, there were no significant changes in tiller number, grain number per spike and spikelet number per spike (Figs. 4E-F, Supplemental Fig. S1). In summary, the overexpression of *TaCR4-A* in wheat significantly increases grain length, width, thickness and weight, and *TaCR4* positively regulates grain size.

**Table 1:** Wheat grain size analysis of KN199 and the *TaCR4-A* OE lines

	KN199	OE1	OE2	OE3
Length(mm)	6.273 ± 0.1385^b^	6.491 ± 0.2056^a^	6.471 ± 0.1597^a^	6.467 ± 0.1361^a^
		(3.48%)	(3.16%)	(3.09%)
Width(mm)	3.814 ± 0.1052^b^	4.069 ± 0.1419^a^	4.057 ± 0.1333^a^	4.051 ± 0.1234^a^
		(6.69%)	(6.37%)	(6.21%)
Thickness(mm)	3.579 ± 0.1428^b^	3.821 ± 0.1632^a^	3.818 ± 0.1426^a^	3.817 ± 0.1376^a^
		(6.76%)	(6.26%)	(6.24%)
Thousand-grain weight(g)	41.27 ± 0.8472^b^	43.57 ± 0.7116^a^	43.53 ± 0.8225^a^	43.45 ± 0.4241^a^
		(5.57%)	(5.48%)	(5.28%)

Samples (*n* = 30) were taken from the middle of the spike for measurement from five independent plants, and the thousand-grain weight was taken from at least 30 plants. The percentage difference is shown in comparison with KN199. Data are means ± SD; different letters indicate statistically significant differences (*P* < 0.05; one-way ANOVA; Tukey’s test)

### TaCR4-A regulates epidermal cell size in wheat grains

**Fig. 5:**
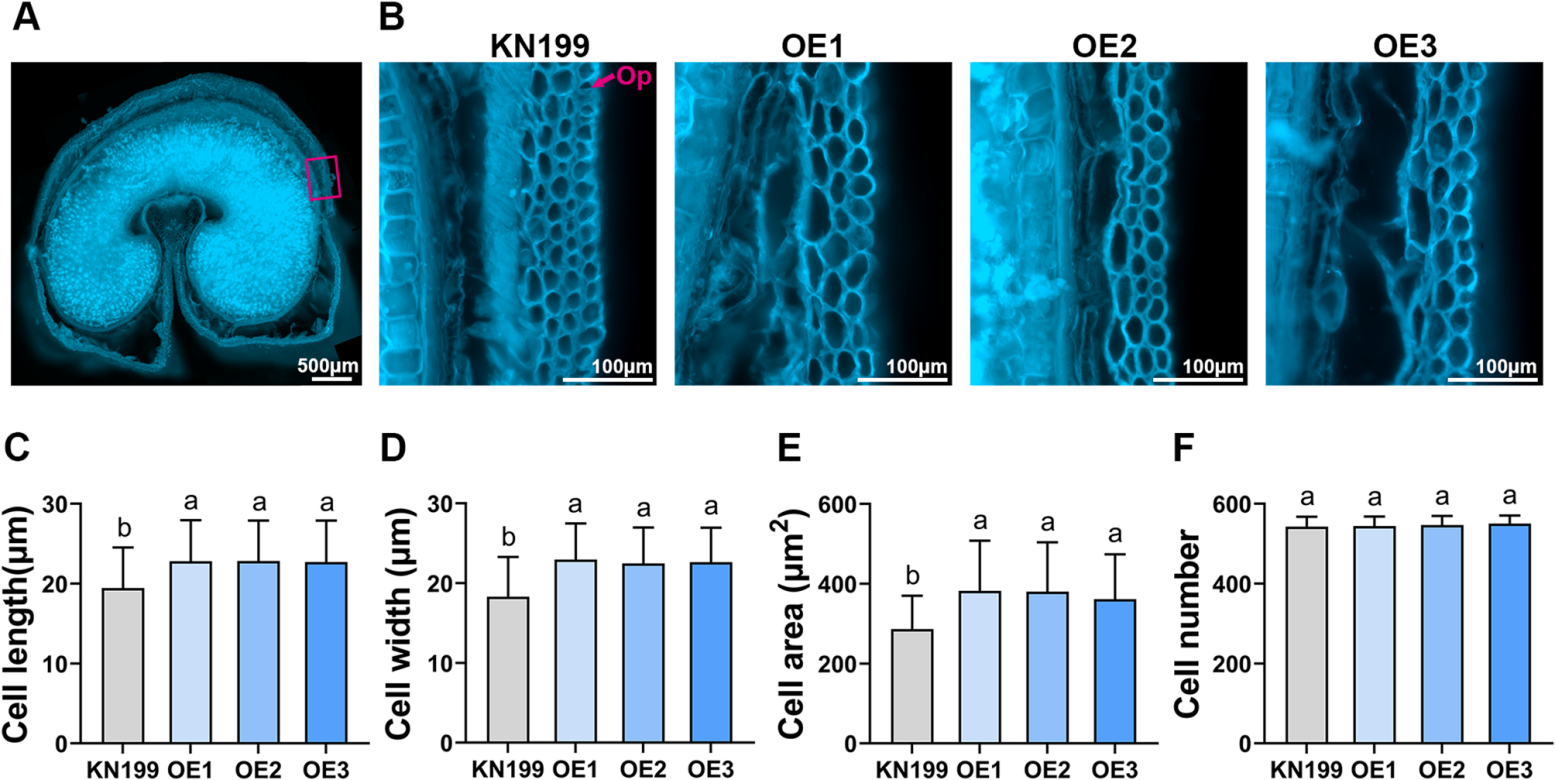
*TaCR4-A* effects wheat grain pericarp cell size. **A** Representative cross section of the middle portion of developing grain size in KN199 at 20DAP. **B** Magnified views of the pericarp cell of the KN199 and *TaCR4* OE lines at the same position as the box region in (**A**). **C**-**F** Quantitative analysis of outer pericarp cell length (**C**), width(**D**), area(**E**), and number(**F**). Cells (*n* = 100) were measured from at least 20 grains taken from five independent plants. Data are means ± SD, different letters indicate statistically significant differences (*P* < 0.05, one-way ANOVA, Tukey’s test). Op: outer pericarp

The size of plant organs is determined by the size and number of their constituent cells. *CR4* has been shown to affect epidermal differentiation and development in several species. To further investigate the cytological basis of wheat grain enlargement in *TaCR4-A* OE lines, we analysed the fluorescently stained seed coat cells in cross sections of developing grains from KN199 and *TaCR4-A* OE lines, and measured the size of the outer pericarp cells (Fig. 5A and B). The results showed that the outer pericarp cells of the *TaCR4-A* OE lines grains were longer, wider and had a larger cell area, while there was no significant difference in the number of outer pericarp cells (Fig. 5C-F). These results suggest that *TaCR4* positively regulates the size of grain epidermal cells, thereby influencing seed coat volume and promoting grain growth, and resulting in larger grains.

## Discussion

Reports have demonstrated that RLKs regulate grain size, with functional variation observed across different subfamilies and species [20–23, 25, 26].*CR4* belongs to a subfamily of the *RLK* gene family in plants and affects the growth and development of plants [39]. It was first reported in maize, and studies on *CR4* homologous genes have since been conducted in multiple plant species [40]. Studies on maize and rice have revealed that it regulates grain size [19, 27]. In this study, we identified the *CR4* homologs encoding genes *TaCR4-A*, *TaCR4-B* and *TaCR4-D* in wheat. These genes are located on wheat chromosomes 7 A, 7B and 7D, respectively, and are continuously expressed during grain filling (Fig. 2). The TaCR4-A protein is composed of 900 amino acids and contains domains such as Pkinase_Tyr and TNFR (Fig. 1), exhibiting 88.25% protein similarity with OsCR4 and 84.5% with CR4 in maize.

*CR4* influences the development of plant seeds [39]. The *CR4* mutation in maize affects leaf epidermis differentiation and inhibits aleurone layer formation in seeds, resulting in crinkly leaves and aleurone defects in small grains [27]. In rice, knocking down *OsCR4* expression leads to a separation of palea and lemma in the spikelet, resulting in damage to the development of the embryo and endosperm, as well as defects in the aleurone [31]. The rice mutant *mis2* (defective in *OsCR4*) has smaller grains, a wrinkled surface and an irregular shape, with reduced length, width, thickness, and thousand-grain weight, and after the complete genomic DNA of *OsCR4* was transformed into the mis2 mutant, the transgenic lines fully complemented the mutant phenotype with large grains [19]. And OsCR4 positively regulates rice culm elongation [41]. In this study, we used the particle bombardment method to overexpress *TaCR4* in the wheat variety KN199. The results indicate that *TaCR4* positively regulates grain size, as the grain length, width, thickness and weight of the overexpressed wheat lines increased significantly (Figs. 3 and 4).

*CR4* plays an essential role in regulating the development and differentiation of plant epidermal cells [30, 39]. Maize *cr4* mutants produce wrinkled and fused leaves due to the *CR4* gene affecting the differentiation of epidermal cells, resulting in changes to cell size and morphology [27]. *ACR4* mutations affect the differentiation of leaf epidermal cells and cause disorganization of cell layers in the integument and endothelium of ovules in Arabidopsis [28, 29, 42]. Knockdown of *OsCR4* in rice affects epidermal cell differentiation, resulting in tumour-like cells on the outer epidermis and wart-like cells on the inner epidermis of spikelets, producing separated palea and lemma [31]. The *mis2* mutant had reduced spikelet epidermal cell size but increased cell number, suggesting that *OsCR4* regulates grain size by coordinately regulating epidermal cell size and cell number [19]. The volume of the seed coat affects grain size by determining the space and potential for grain development [43]. To examine the cellular basis of grain size variation, we collected developing grains at 20 DAP and analyzed the seed coat cell size in cross sections. The results showed that the seed coat cell size of the *TaCR4*-OE lines was larger than KN199. We quantified and statistically analyzed the length, width and area of the pericarp cells, finding that they had increased significantly, while there was no significant difference in cell number (Fig. 5). Our study shows that *TaCR4* affects grain size by regulating the size of seed coat cells by influencing their differentiation and development, but that the mechanism of *TaCR4* action requires further research.

## Conclusions

This study identified the *TaCR4* gene in wheat, which encodes the Crinkly receptor-like kinase homologue, and demonstrated that it positively regulates wheat grain size. Overexpressing *TaCR4-A* in the wheat variety KN199 significantly increased the grain length, width, thickness and thousand-grain weight. Cytological analysis revealed that the length, width and area of the outer epidermal cells of the developing grains in the overexpressed wheat lines had increased. This suggests that the overexpression of *TaCR4-A* influences grain size by regulating the size of grain epidermal cells. The study of *TaCR4* could provide potential applications in improving wheat breeding.

## Supplementary Information

Supplementary Material 1. Figure S1. Agronomic traits of TaCR4-A OE wheat lines. Table S1. Primers used in this study. Supplementary Data 1. Sequence of CR4 homologous proteins in different species.

## Data Availability

All data supporting the findings of this study are available within the article and its supplementary data, or are available from the corresponding author on reasonable request.

## References

[1] Liu Y, Wang M, Wang Y, Liu H, Xi W, Seung D, et al. The transcription factor MYB44 suppresses starch synthesis to negatively regulate grain weight and yield in wheat and rice. Mol Plant. 2025. 10.1016/j.molp.2025.06.007. 40534131 10.1016/j.molp.2025.06.007

[2] Yun L, Chen T, Chen M, Wang P, Tian T, Zhang Y, et al. The wheat fructokinase gene *TaFRK2-7B1* positively regulates grain size by modulating fructose metabolism. Int J Biol Macromol. 2025;318:144939. 10.1016/j.ijbiomac.2025.144939. 40473157 10.1016/j.ijbiomac.2025.144939

[3] Qian Q, Hu W, Bi Z, Li L, Wang K, Zhang X, et al. The cytokinin oxidase/dehydrogenase TaCKX11-D positively regulates grain size in wheat. J Exp Bot. 2025;eraf196. 10.1093/jxb/eraf196. 10.1093/jxb/eraf196 40357640

[4] Phillips AL, Huttly AK, Alarcón-Reverte R, Clark SJ, Jaworek P, Tarkowská D, et al. GIBBERELLIN 3-OXIDASE genes regulate height and grain size in bread wheat. J Exp Bot. 2025;76:3345–58. 10.1093/jxb/eraf151. 40208256 10.1093/jxb/eraf151

[5] Zhang Y, Liu H, Wang Y, Si X, Pan Y, Guo M, et al. TaFT-D1 positively regulates grain weight by acting as a coactivator of TaFDL2 in wheat. Plant Biotechnol J. 2025;23:2207–23. 10.1111/pbi.70032. 40100647 10.1111/pbi.70032 PMC12120878

[6] Liu H, Li T, Hou J, Yin X, Wang Y, Si X, et al. Tawus-like-5D affects grain weight and filling by inhibiting the expression of sucrose and trehalose metabolism-related genes in wheat grain endosperm. Plant Biotechnol J. 2025;23:2018–33. 10.1111/pbi.70015. 40048350 10.1111/pbi.70015 PMC12120876

[7] Li B, Jiang X, Chai Z, Liu J, Gao C, Chen K. Disruption of the miR396 binding site in GROWTH-REGULATING FACTOR 4 enhances grain size in wheat. Sci China Life Sci. 2025;68:2170–2. 10.1007/s11427-024-2878-9. 40048069 10.1007/s11427-024-2878-9

[8] Wang W, Pan Q, Tian B, Yu Z, Davidson D, Bai G, et al. Non-additive dosage-dependent effects of *TaGS3* gene editing on grain size and weight in wheat. Theor Appl Genet. 2025;138:38. 10.1007/s00122-025-04827-w. 39880939 10.1007/s00122-025-04827-w PMC11779757

[9] Yan L, Jiao B, Duan P, Guo G, Zhang B, Jiao W, et al. Control of grain size and weight by the RNA-binding protein EOG1 in rice and wheat. Cell Rep. 2024;43:114856. 10.1016/j.celrep.2024.114856. 39427319 10.1016/j.celrep.2024.114856

[10] Liu H, Li H, Hao C, Wang K, Wang Y, Qin L, et al. TaDA1, a conserved negative regulator of kernel size, has an additive effect with TaGW2 in common wheat (*Triticum aestivum* L). Plant Biotechnol J. 2020;18:1330–42. 10.1111/pbi.13298. 31733093 10.1111/pbi.13298 PMC7152612

[11] Ma L, Li T, Hao C, Wang Y, Chen X, Zhang X. TaGS5-3A, a grain size gene selected during wheat improvement for larger kernel and yield. Plant Biotechnol J. 2016;14:1269–80. 10.1111/pbi.12492. 26480952 10.1111/pbi.12492 PMC11389196

[12] Simmonds J, Scott P, Brinton J, Mestre TC, Bush M, del Blanco A, et al. A splice acceptor site mutation in TaGW2-A1 increases thousand grain weight in tetraploid and hexaploid wheat through wider and longer grains. Theor Appl Genet. 2016;129:1099–112. 10.1007/s00122-016-2686-2. 26883045 10.1007/s00122-016-2686-2 PMC4869752

[13] Su Z, Hao C, Wang L, Dong Y, Zhang X. Identification and development of a functional marker of TaGW2 associated with grain weight in bread wheat (*Triticum aestivum* L). Theor Appl Genet. 2011;122:211–23. 10.1007/s00122-010-1437-z. 20838758 10.1007/s00122-010-1437-z

[14] Zhang L, Zhao Y-L, Gao L-F, Zhao G-Y, Zhou R-H, Zhang B-S, et al. TaCKX6-D1, the ortholog of rice OsCKX2, is associated with grain weight in hexaploid wheat. New Phytol. 2012;195:574–84. 10.1111/j.1469-8137.2012.04194.x. 22670578 10.1111/j.1469-8137.2012.04194.x

[15] Shiu S-H, Bleecker AB. Plant receptor-like kinase gene family: diversity, function, and signaling. Science’s STKE. 2001;2001:re22-22. 10.1126/stke.2001.113.re22. 11752632 10.1126/stke.2001.113.re22

[16] Shiu S-H, Bleecker AB. Receptor-like kinases from Arabidopsis form a monophyletic gene family related to animal receptor kinases. Proc Natl Acad Sci U S A. 2001;98:10763–8. 10.1073/pnas.181141598. 11526204 10.1073/pnas.181141598 PMC58549

[17] Becraft PW. Receptor kinases in plant development. Trends Plant Sci. 1998;3:384–8. 10.1016/S1360-1385(98)01301-6.

[18] De Smet I, Voß U, Jürgens G, Beeckman T. Receptor-like kinases shape the plant. Nat Cell Biol. 2009;11:1166–73. 10.1038/ncb1009-1166. 19794500 10.1038/ncb1009-1166

[19] Chun Y, Fang J, Zafar SA, Shang J, Zhao J, Yuan S, et al. MINI SEED 2 (MIS2) encodes a receptor-like kinase that controls grain size and shape in rice. Rice. 2020;13:7. 10.1186/s12284-020-0368-9. 32006119 10.1186/s12284-020-0368-9 PMC6994593

[20] Yuan H, Xu Z, Chen W, Deng C, Liu Y, Yuan M, et al. OsBSK2, a putative brassinosteroid-signalling kinase, positively controls grain size in rice. J Exp Bot. 2022;73:5529–42. 10.1093/jxb/erac222. 35595300 10.1093/jxb/erac222

[21] Chen C, Hu X, Du H, Dong Q, Fu Y, Li Y, et al. S-domain receptor-like protein kinase OsGRSK1 participates in regulating plant height and grain size via Gibberellin pathway in rice. Plant Sci. 2025;358:112579. 10.1016/j.plantsci.2025.112579. 40414360 10.1016/j.plantsci.2025.112579

[22] Zhao K, Wang L, Qiu D, Cao Z, Wang K, Li Z, et al. PSW1, an LRR receptor kinase, regulates pod size in peanut. Plant Biotechnol J. 2023;21:2113–24. 10.1111/pbi.14117. 37431286 10.1111/pbi.14117 PMC10502750

[23] He C, Wang J, Dong R, Guan H, Liu T, Liu C, et al. Overexpression of an antisense RNA of maize receptor-like kinase gene ZmRLK7 enlarges the organ and seed size of transgenic Arabidopsis plants. Front Plant Sci. 2020. 10.3389/fpls.2020.579120. 33304362 10.3389/fpls.2020.579120 PMC7693544

[24] Singh A, Khurana P. Ectopic expression of *triticum aestivum* SERK genes (TaSERKs) control plant growth and development in Arabidopsis. Sci Rep. 2017;7:12368. 10.1038/s41598-017-10038-1. 28959050 10.1038/s41598-017-10038-1 PMC5620050

[25] Xiao W, Hu S, Zou X, Cai R, Liao R, Lin X, et al. Lectin receptor-like kinase LecRK-VIII.2 is a missing link in MAPK signaling-mediated yield control. Plant Physiol. 2021;187:303–20. 10.1093/plphys/kiab241. 34618128 10.1093/plphys/kiab241 PMC8418426

[26] Wu X, Cai X, Zhang B, Wu S, Wang R, Li N, et al. ERECTA regulates seed size independently of its intracellular domain via MAPK-DA1-UBP15 signaling. Plant Cell. 2022;34:3773–89. 10.1093/plcell/koac194. 35848951 10.1093/plcell/koac194 PMC9516062

[27] Becraft PW, Stinard PS, McCarty DR. CRINKLY4: a TNFR-like receptor kinase involved in maize epidermal differentiation. Science. 1996;273:1406–9. 10.1126/science.273.5280.1406. 8703079 10.1126/science.273.5280.1406

[28] Watanabe M, Tanaka H, Watanabe D, Machida C, Machida Y. The ACR4 receptor-like kinase is required for surface formation of epidermis-related tissues in *Arabidopsis thaliana*. Plant J. 2004;39:298–308. 10.1111/j.1365-313X.2004.02132.x. 15255860 10.1111/j.1365-313X.2004.02132.x

[29] Cao X, Li K, Suh S-G, Guo T, Becraft PW. Molecular analysis of the CRINKLY4 gene family in *Arabidopsis thaliana*. Planta. 2005;220:645–57. 10.1007/s00425-004-1378-3. 15549374 10.1007/s00425-004-1378-3

[30] Tanaka H, Watanabe M, Watanabe D, Tanaka T, Machida C, Machida Y. ACR4, a putative receptor kinase gene of *Arabidopsis thaliana*, that is expressed in the outer cell layers of embryos and plants, is involved in proper embryogenesis. Plant Cell Physiol. 2002;43:419–28. 10.1093/pcp/pcf052. 11978870 10.1093/pcp/pcf052

[31] Pu C-X, Ma Y, Wang J, Zhang Y-C, Jiao X-W, Hu Y-H, et al. Crinkly4 receptor-like kinase is required to maintain the interlocking of the palea and lemma, and fertility in rice, by promoting epidermal cell differentiation. Plant J. 2012;70:940–53. 10.1111/j.1365-313X.2012.04925.x. 22332708 10.1111/j.1365-313X.2012.04925.x

[32] IWGSC. Shifting the limits in wheat research and breeding using a fully annotated reference genome. Science. 2018;361:eaar7191. 10.1126/science.aar7191. 30115783 10.1126/science.aar7191

[33] Letunic I, Khedkar S, Bork P. SMART: recent updates, new developments and status in 2020. Nucleic Acids Res. 2021;49:D458–60. 10.1093/nar/gkaa937. 33104802 10.1093/nar/gkaa937 PMC7778883

[34] Tamura K, Stecher G, Kumar S. MEGA11: Molecular Evolutionary Genetics Analysis Version 11. Mol Biol Evol. 2021;38:3022–7. 10.1093/molbev/msab120. 33892491 10.1093/molbev/msab120 PMC8233496

[35] Livak KJ, Schmittgen TD. Analysis of relative gene expression data using Real-Time quantitative PCR and the 2 – ∆∆CT method. Methods. 2001;25:402–8. 10.1006/meth.2001.1262. 11846609 10.1006/meth.2001.1262

[36] Xiao J, Xu S, Li C, Xu Y, Xing L, Niu Y, et al. O-GlcNAc-mediated interaction between VER2 and TaGRP2 elicits TaVRN1 mRNA accumulation during vernalization in winter wheat. Nat Commun. 2014;5:4572. 10.1038/ncomms5572. 25091017 10.1038/ncomms5572 PMC4143922

[37] Ursache R, Andersen TG, Marhavý P, Geldner N. A protocol for combining fluorescent proteins with histological stains for diverse cell wall components. Plant J. 2018;93:399–412. 10.1111/tpj.13784. 29171896 10.1111/tpj.13784

[38] Thévenaz P, Unser M. User-friendly semiautomated assembly of accurate image mosaics in microscopy. Microsc Res Tech. 2007;70:135–46. 10.1002/jemt.20393. 17133410 10.1002/jemt.20393

[39] Czyzewicz N, Nikonorova N, Meyer MR, Sandal P, Shah S, Vu LD, et al. The growing story of (ARABIDOPSIS) CRINKLY 4. J Exp Bot. 2016;67:4835–47. 10.1093/jxb/erw192. 27208540 10.1093/jxb/erw192

[40] Nikonorova N, Vu LD, Czyzewicz N, Gevaert K, De Smet I. A phylogenetic approach to study the origin and evolution of the CRINKLY4 family. Front Plant Sci. 2015;6. 10.3389/fpls.2015.00880. 10.3389/fpls.2015.00880 PMC4617170 26557128

[41] Pu C-X, Sun Y. Rice Crinkly4 receptor-like kinase positively regulates culm elongation and amino acid K532 is not essential for its kinase activity. Plant Signal Behav. 2012;7:1062–4. 10.4161/psb.21106. 22899082 10.4161/psb.21106 PMC3489627

[42] Gifford ML, Dean S, Ingram GC. The Arabidopsis ACR4 gene plays a role in cell layer organisation during ovule integument and sepal margin development. Development. 2003;130:4249–58. 10.1242/dev.00634. 12900442 10.1242/dev.00634

[43] Li N, Xu R, Li Y. Molecular Networks of Seed Size Control in Plants. Annual Review of Plant Biology. 2019;70 Volume 70, 2019:435–63. 10.1146/annurev-arplant-050718-095851 10.1146/annurev-arplant-050718-095851 30795704

