# Supplementary material for: Overexpression of TaCR4-A positively regulates grain size in Triticum aestivum
Source: BMC Plant Biol. 2025 Oct 1;25:1273. doi: 10.1186/s12870-025-07345-5 (PMC12487043; doi:10.1186/s12870-025-07345-5)
Supplement: Supplementary file 1 — Supplementary Material 1. Figure S1. Agronomic traits of TaCR4-A OE wheat lines. Table S1. Primers used in this study. Supplementary Data 1. Sequence of CR4 homologous proteins in different species. [file 12870_2025_7345_MOESM1_ESM.docx]

**Supplemental Data**

**
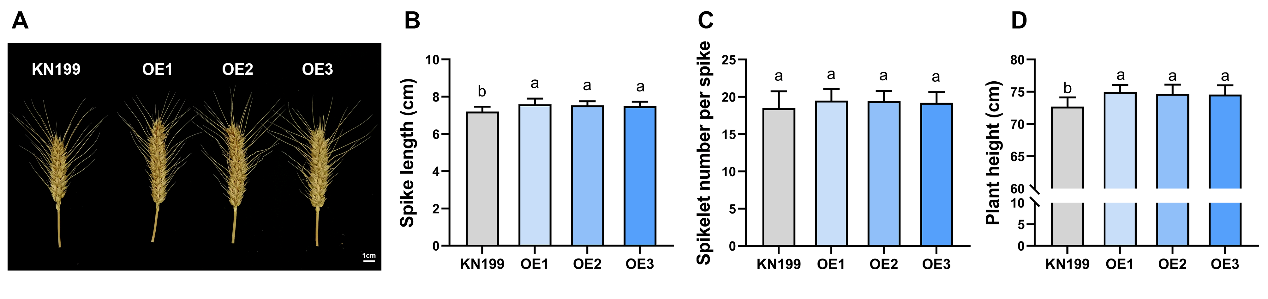
Supplemental Figure S1.** **Agronomic traits of *TaCR4-A* OE wheat lines.**

(A) Mature spike of KN199 and *TaCR4-A* OE lines, bars = 1 cm.

(B-D) Measurement of spike length, spikelet number per spike, and plant height of WT and *TaCR4-A* OE lines. Data are means ± SD (n = 20), different letters indicate statistically significant differences, (P < 0.05, one-way ANOVA, Tukey's test).

**Supplemental Table S1. Primers used in this study.**

| Primer | Primer Sequence (5' to 3') Purpose | Purpose |
| --- | --- | --- |
| TaCR4-A-F | TGCTCTTCCCGAGGTATCCG | TaCR4-A open reading frame cloning |
| TaCR4-A-R | TGTTTCCAGCTGATCTTGGCA |  |
| pWMB003-TaCR4-A-F | CCCCGGGTACCGAGCTC ATCTCACCGACATTAGCTCCG | Transgenic wheat |
| pWMB003-TaCR4-A-R | CGGGGAAATTCGAGCTC GAAGTTGTGCTGCAAGTAC |  |
| TaCR4-A-qPCR-F | TGATTGAGCTGGCTCCGAGG | qRT-PCR |
| TaCR4-A-qPCR-R | CCTGATGCCACAAGCATGGA |  |
| TaActin-qPCR-F | TGCTATCCTTCGTTTGGACCTT | qRT-PCR |
| TaActin-qPCR-R | AGCGGTTGTTGTGAGGGAGT |  |
| Test-transform-F | ACATCCATTTAGGGTTTAGGGTT | Transgenic wheat detection |
| Test-transform-R | CGCAATTTCTGGATGCCGAC |  |

**Supplementary Data 1. Sequence of CR4 homologous proteins in different species.**

>*TaCR4-A* TraesCS7A02G301300.1

MDSVPALALCCLILLPSWANGLGSMGSISVSYGEDGPVFCGLSSDGSHLVTCSGADASVVYGAPLRIPFLGLTAGDGFACGLLLDTSQPYCWGSNSYVKIGVPQPMVEGVEYSLLSAGDNHLCALRMPDKGIPHGVNPDTSVIDCWGYNMTATHVVAGAVSTISAGSVFNCALFTRNRTVFCWGDETVSGVIELAPRNVKFQSIGAGGYHVCGVLENAQVFCWGRSLEMQQVSPTGAIGEGDVSIVPMDAMVSVVGGRFHACGIRSLDHQVACWGFQLQNSTSAPKGLRLYTIVAGDYFTCGVPAETSMKPRCWGNSGPLALPMAVSPGICVSSACSPGYYEYVNHGELGGSKSCKPGNSRLCLPCSAGCPDNSYESSPCNATADRVCQFDCSRCVSDECLSYCTSRKQTNNHKSMDFQMRIFVAEIAFAVILIFTVTAISCLYVRHKLRDCRCSKSKLRMTKSATYSFRKDNTKIQPDVEDLKIRRAQEFSYEELEQATDGFSEDSQVGKGSFSCVFRGILRDGTVVAVKRAIKVSDAKKSSKEFHTELDLLSRLNHAHLLDLLGYCEDGSERLLVYEFMAHGSLYQHLHGKDSNLKKQLNWTRRVTIAVQAARGIEYLHGYACPPVIHRDIKSSNILIDEDHNARVADFGLSIMGPVDSGTPLSELPAGTLGYLDPEYYRLHYLTTKSDVYSFGVVLLEILSGRKAIDMQLEEGNIVEWAAPLIKAGDISGILDPALSPPSDLEALKKIAAVACKCVRMRGKDRPSMDKVTTSLERALALLMGSPCLEQPILPTEVVLGSSRMHKKVSQRSSNQSCSENELVDGDDQRIEYRAPSWITFPSVTSSQRRKSSASEADLDGRTTTDGRNVGSSIGDGLRSLEEEIGPASPQEDLYLQHNF

>*TaCR4-B* TraesCS7B02G201600.1

MDSIPALALCCLILLPSWANGLGSMGSISVSYGEDGPVFCGLSSDGSHLVACSGADASVVYGAPLRIPFLGLTAGDGFACGLLLDTSQPYCWGSNSHVKIGVPQPMVEGVEYSMLSAGDNHLCALRMPDKGIPHGVNPDTSVIDCWGYNMTATHVVAGAVSTISAGSVFNCALFARNRTVFCWGDETVSGVIGLAPRNVKFQSIGAGGYHVCGVLENAQVFCWGRSLEMQQVSPTGAIGEGDVSIVPMDAMVSVVGGRFHACGIRSLDHQVACWGFQLQNSTSAPKGLRLYAIVAGDYFTCGVPAETSMKPRCWGNSGPLALPMAVSPGICVSSACSPGYYEYVNHGELGSSKSCKPGNSRLCLPCSAGCPDNSYESSPCNATADRVCQFDCSRCVSDECLSYCTSRKQTNNHKSMDFQMRIFVAEIAFAVILIFTVTAISCLYVRHKLRDCRCSKSKLRMTKSATYSFRKDNTKIQPDVGDLKIRRAQEFSYEELEQATDGFSEDSQVGKGSFSCVFRGILRDGTVVAVKRAIKVSDAKKSSKEFHTELDLLSRLNHAHLLDLLGYCEDGSERLLVYEFMAHGSLYQHLHGKDSNLKKQLNWTRRVTIAVQAARGIEYLHGYACPPVIHRDIKSSNILIDEDHNARVADFGLSIMGPVDSGTPLSELPAGTLGYLDPEYYRLHYLTTKSDVYSFGVVLLEILSGRKAIDMQLEEGNIVEWAAPLIKAGDISGILDPALSPPSDLEALKKIAAVACKCVRMRGKDRPSMDKVTTSLERALALLMGSPCLEQPILPTEVVLGSSRMHKKVSQRSSNQSCSENELVDGDDQRIEYRAPSWITFPSVTSSQRRKSSASEADLDGRTTTDGRNVGSSIGDGLRSLEEEIGPASPQEDLYLQHNF

>*TaCR4-D* TraesCS7D02G296700.1

MDSVPALALCCLILLPSWANGLGSMGSISVSYGEDGPVFCGLSSDGSHLVTCSGADASVVYGAPLRIPFLGLTAGDGFACGLLLDTSQPYCWGSNSYVKIGVPQPMVEGVEYSMLSAGDNHLCALRTPDKGIPRGVNPDTSVIDCWGYNMTATHVVAGAVSTISAGSVFNCALFARNRTVFCWGDETVSGVIGLAPRNVKFQSIGAGGYHVCGVLENAQVFCWGRSLEMQQVSPTGAIGEGDVSIVPMDAMVSVVGGRFHACGIRSLDHQVACWGFQLQNSTSAPKGLRLYTIVAGDYFTCGVPAETSMKPRCWGNSGPLALPMAVSPGICVSSACSPGYYEYVNHGELGSSKSCKPGNSRLCLPCSAGCPDNSYESSPCNATADRVCQFDCLRCVSDECLSYCTSRKQTNNHKSMDFQMRIFVAEIAFAVILIFTVTAISCLYVRHKLRDCRCSKSKLRMTKSTTYSFRKDNTKIQPDVEDLKIRRAQEFSYEELEQATDGFSEDSQVGKGSFSCVFRGILRDGTVVAVKRAIKVSDAKKSSKEFHTELDLLSRLNHAHLLDLLGYCEDGSERLLVYEFMAHGSLYQHLHGKDSNLKKQLNWTRRVTIAVQAARGIEYLHGYACPPVIHRDIKSSNILIDEDHNARVADFGLSIMGPVDSGTPLSELPAGTLGYLDPEYYRLHYLTTKSDVYSFGVVLLEILSGRKAIDMQLEEGNIVEWAAPLIKAGDISGILDPALSPPSDLEALKKIAAVACKCVRMRGKDRPSMDKVTTSLERALALLMGSPCLEQPILPTEVVLGSSRMHKKVSQRSSNQSCSENELVDGDDQRIEYRAPSWITFPSVTSSQRRKSSASEADLDGRTTTDGRNVGSSIGDGLRSLEEEIGPASPQEDLYLQHNF

>*Arabidopsis thaliana* AT3G59420.1

MRMFETRAREWILLVKLVLFTSIWQLASALGSMSSIAISYGEGGSVFCGLKSDGSHLVVCYGSNSAILYGTPGHLQFIGLTGGDGFMCGLLMLSHQPYCWGNSAFIQMGVPQPMTKGAEYLEVSAGDYHLCGLRKPIVGRRKNSNIISSSLVDCWGYNMTRNFVFDKQLHSLSAGSEFNCALSSKDKSVFCWGDENSSQVISLIPKEKKFQKIAAGGYHVCGILDGLESRVLCWGKSLEFEEEVTGTSTEEKILDLPPKEPLLAVVGGKFYACGIKRYDHSAVCWGFFVNRSTPAPTGIGFYDLAAGNYFTCGVLTGTSMSPVCWGLGFPASIPLAVSPGLCIDTPCPPGTHELSNQENSPCKFTGSHICLPCSTSCPPGMYQKSVCTERSDQVCVYNCSSCSSHDCSSNCSSSATSGGKEKGKFWSLQLPIATAEIGFALFLVAVVSITAALYIRYRLRNCRCSENDTRSSKDSAFTKDNGKIRPDLDELQKRRRARVFTYEELEKAADGFKEESIVGKGSFSCVYKGVLRDGTTVAVKRAIMSSDKQKNSNEFRTELDLLSRLNHAHLLSLLGYCEECGERLLVYEFMAHGSLHNHLHGKNKALKEQLDWVKRVTIAVQAARGIEYLHGYACPPVIHRDIKSSNILIDEEHNARVADFGLSLLGPVDSGSPLAELPAGTLGYLDPEYYRLHYLTTKSDVYSFGVLLLEILSGRKAIDMHYEEGNIVEWAVPLIKAGDINALLDPVLKHPSEIEALKRIVSVACKCVRMRGKDRPSMDKVTTALERALAQLMGNPSSEQPILPTEVVLGSSRMHKKSWRIGSKRSGSENTEFRGGSWITFPSVTSSQRRKSSASEGDVAEEEDEGRKQQEALRSLEEEIGPASPGQSLFLHHNF

>*Glycine max* GLYMA_12G052300

MSKQFCYYYWSIFYILHFFLCVIFWLLCIMGFSLKHRLIYGFNVNQCSTRLLFELVILSHLWLQVTSLGSMSSIAISYGEKGSVFCGLKSDGSHTVTCYGSNSAIIYGTPTHFSFLGLTAGDGFVCGLLMGSNQPYCWGSSAYIEMGVPQPMIKGAQYLEISAGDYHVCGLRKPMTGRHRNISLVDCWGYNMTKNYVFGAQIQSISAGSEFNCGLFSQNRTVFCWGDETNSLVISLIPHDMRFHKISAGGYHVCGISEGVSSKTFCWGRSLNLEEEISVSHAGQGNVDLAPNDPMLSVVGGKFHACGIKSYDRGVICWGFIIKPSTPSPKGIKVFEVAAGDYFTCAVLAVKSLMPSCWGVDFPTSLPLAVSPGMCQPAPCAPGSYAIDQHKSLCKSPDSRVCMRCSGACPPEMHLKSACNLASDRVCEYNCSCCSSSECFLNCSSSYSNAAAAEKKSEKFWALQLPVLIAEIAFAVFVVSIVSITAVLYIRYRLRDCECSKGSMVKKLNGNSSLQNENKVRPDLEELKIRRAQTFTYEELETATSGFKEESIVGKGSFSCVFKGVLKDGTVVAVKRAIVSPNMQKNSKEFHTELDLLSRLNHAHLLNLLGYCEEGGERLLVYEFMAHGSLHQHLHATNQVLREQLDWIRRVTIAVQAARGIEYLHGYACPPVIHRDIKSSNILIDEEHNARVADFGLSLLGPADSGSPLAELPAGTLGYLDPEYYRLHYLTTKSDVYSFGVLLLEILSGRKAIDMQYEEGNIVEWAVPLIKSGDITAILDPVLKPPPDLEALKRIANVACKCVRMRGKERPSMDKVTTALERGLAQLMGSPCIEQPILPTEVVLGSNRLHKKSSQRSSNRSVSETDVAETEDQRFEFRAPSWITFPSVTSSQRRKSSVSEADVDGKNNAEGKNMGNVGGGGDVLRSLEEEIGPASPRERLFLQHNF

>*Hordeum vulgare* XP_044956830.1

MGSVLALSLCCLTLLPSWAYGLGSMGSISVSYGEDGPVFCGLSSDGSHLVACSGADASVVYGAPLRIPFLGLTAGDGFACGLLLDTSQPYCWGSNSYVKIGVPQPMVEGVEYSMLSAGDNHLCALRMPDKGIPHGDTSVIDCWGYNMTATHVVAGAVSTISAGSVFNCALFSRNRTVFCWGDETVSGVIGLAPRNVKFQSIGAGGYHVCGVLENAQVFCWGRSLEMQQVSPTGAIGEGDVSIVPMDAMVSVVGGRFHACGIRSLDHQVACWGFQLQNSTSAPKGLRLYAIVAGDYFTCGVPAETSMKPRCWGNSGPLALPMAVSPGICVSAACSPGYYEYVNHGELGGSKACKPGNSRLCLPCSAACPDNSYESSPCNVTADRVCQFDCSRCVSDECWSYCTSRKQTNNHKSMDFQMHIFVAEIAFAIILIFTVTAIACLYVRHKLRDCRCSKSKLRMTKSTTYSFRKDNMKIQPDVEDLKIRRAQEFSYEELEQATDGFSEDSQVGKGSFSCVFRGILRDGTVVAVKRAIKVSDAKKSSKEFHTELDLLSRLNHAHLLDLLGYCEDGSERLLVYEFMAHGSLYQHLHGKDSNLKKQLNWTRRVTIAVQAARGIEYLHGYACPPVIHRDIKSSNILIDEDHNARVADFGLSIMGPVDSGTPLSELPAGTLGYLDPEYYRLHYLTTKSDVYSFGVVLLEILSGRKAIDMQLEEGNIVEWAAPLIKAGDISGILDPALSPPSDPEALKKIAAVACKCVRMRGKDRPSMDKVTTSLERALALLMGSPCIEQPILPTEVVLGSSRMHKKVSQRSSNQSCSENELVDGDDQRIEYRAPSWITFPSVTSSQRRKSSASEADLDGRTTTDGRNVGSSIGDGLRSLEEEIGPASPQENLYLQHNF

>*Oryza sativa Japonica Group* LOC_Os03g43670.1

MDIVPVVALCCCLVLLPSWAYGLGSMASIAVSYGEDGPVFCGLNSDGSHLVTCFGADASVVYGAPSRIPFVGVTAGDGFACGLLLDTNQPYCWGSNSYVKIGVPQPMVEGAMYSELSAGDNHLCALRTSVKGFHSVNGDTSVIDCWGYNMTATHTVTGAVSAISAGSVFNCGLFARNRTVFCWGDESVSGVIGLAPRNVRFQSIGAGGYHVCGVLENAQVFCWGRSLEMQQMSTPSSTDDGDVNIVPMDAMVSVVGGRFHACGIRSLDHQVACWGFTLQNSTLAPKGLRVYAIVAGDYFTCGVPAETSLKPMCWGHSGPLALPMAVSPGICVSDSCSHGYYEYANHGEVGSGSKTCKPANSRLCLPCSVGCPDDSYESSPCNATADRVCQFDCSKCASDECVSFCLSQKRTKNRKFMAFQLRIFVAEIAFAVILVFSVTAIACLYVRYKLRHCQCSKNELRLAKNTTYSFRKDNMKIQPDVEDLKIRRAQEFSYEELEQATGGFSEDSQVGKGSFSCVFKGILRDGTVVAVKRAIKASDVKKSSKEFHTELDLLSRLNHAHLLNLLGYCEDGSERLLVYEFMAHGSLYQHLHGKDPNLKKRLNWARRVTIAVQAARGIEYLHGYACPPVIHRDIKSSNILIDEDHNARVADFGLSILGPADSGTPLSELPAGTLGYLDPEYYRLHYLTTKSDVYSFGVVLLEILSGRKAIDMQFEEGNIVEWAVPLIKAGDISALLDPVLSPPSDLEALKKIAAVACKCVRMRAKDRPSMDKVTTALERALALLMGSPCIEQPILPTEVVLGSSRMHKKVSQRSSNHSCSENDLVDGDDQRIEYRAPSWITFPSVTSSQRRKSSASEADMDGRTTTDGRNVGSSIGDGLRSLEEEISPASPQENLYLQHNF

>*Sorghum bicolor* SORBI_3008G038200

MDHVPALVLAAVCFLALLPGWASGLGSMSSIAVSYGEDGPVFCGLNSDGSHLVACFGADASVLYGAPPNIPFLGLTAGDGFVCGLLLDTRQPYCWGSNSYVKSGVPQPMVEGARYSELSAGDNHLCALRVAEDGGRGSSAASAKALIDCWGYNMTATHVVDEAVSTVSAGSVFNCGLFARNRTVFCWGDETVSGVVGLAPRNVRFQSIGAGGYHVCGVLENAQVFCWGRSLEMQQVAPSSAIGVGDVNIVPMDAMVAVVGGRFHACGIRSLDHQVACWGFTLHNSTSPPKGLKMYALVAGDYFTCGVPAETSLMPRCWGNSGPLALPMAVPPGICVPTACSHGYYEYVNHGEVGSIKVCKPANSRLCLPCSTGCPEDSYESSPCNATADHVCQFDCSRCGTDECLSFCLSQKRTKSHKLMAFQMRIFVAEIVFAIILVLSVSVISCLYVRHKLRHCQCSNRELRLAKSTAYSFRKDNMRIQPDVEDLKIRRAQVFSYEELEQATGGFSEDSQVGKGSFSCVFKGILRDGTVVAVKRAIKASDVKKSSKEFHNELDLLSRLNHAHLLNLLGYCEDGSERLLVYEFMAHGSLYQHLHGKDPNLKKRLNWARRVTIAVQAARGIEYLHGYACPPVIHRDIKSSNILIDEDHNARVADFGLSILGPADSGTPLSELPAGTLGYLDPEYYRLHYLTTKSDVYSFGVVLLEILSGRKAIDMQFEEGNIVEWAVPLIKAGDIFAILDPALSPPSDLEALKKIASVACKCVRMRGKDRPSMDKVTTALEHALALLMGSPCIEQPILPTEVVLGSSRMHKVSQMSSNHSCSENELADGEDQRIEYRAPSWITFPSVTSSQRRKSSASEADIVGRRTTDGRNVGSSIGDGLRSLEEEIAPASPQENLYLQHNF

>*Vitis vinifera* Vitis05g01875

MGISRSLLEHFLNWVLKIQTWQAVFLVQIRVLVVFSNLWWLVSGLGSMSSIAISYGENGPVFCGLKSDGSHLVTCYGSNSAIIYGTPAHFPFMGLTAGDGFVCGLLVDSNQPYCWGSSRYVQMGVPQPMIKGAEYLEISAGDYHLCGLREPLTGRLRNYSLVDCWGYNMTRSYRFDGQLQSISAGSEFNCGLFSQNRTVFCWGDETSSRVTSLIPQEMRFQKIAAGGYHVCGILEGANSRVFCWGGRSLDIEEEISTAYTGQGNVDSAPKDPMLSVVGGKFHACGIRSSDRGVTCWGFRVKTSTLPPDGIKVYEIAAGNYFTCGILAEKSLLPVCWGLGFPSSLPLAVSPGLCTPSPCLPGFYEFNHESPPCKSLNSHVCLPCSSACLDDMYQKAECTLKSDRQCEFNCSGCYSAECFSNCSSSSYANAITGRKTERFWSLQLPVVVAEVAFAVFLVSIVSLTTILYVRYKLRNCRCSDKGLKSKKGKANGSSFQNDNSKIRPDLDELKIRRAQTFTYDELERATGGFKEESQVGKGSFSCVFKGVLKDGTVVAVKRATMSSDMKKNSKEFHTELDLLSRLNHAHLLNLLGYCEEGGERLLVYEFMAHGSLHQHLHGKNKALKEQLDWVRRVTIAVQAARGIEYLHGYACPPVIHRDIKSSNILIDEEHNARVADFGLSLLGPADSGSPLAEPPAGTFGYLDPEYYRLHYLTTKSDVYSFGVLLLEILSGRKAIDMQFDEGNIVEWAVPLIKSGDISAILDPVLKPPSDLEALKRIATVAYKCVRMRGKERPSMDKVTTALERALAQLMGSPCNEQPILPTEVVLGSSRLHKKSSQRSSNRSVSETDVAEAEDQRFEFRAPSWITFPSVASSQRRKSSVSEADVDGKNLEARNLGSGGNGGDGLRSLEEEIGPASPQENLFLQHNF

>*Zea mays* Zm00001eb406780

MDHVPALVLAGCCFLALLPGWACGLGSMSSIAVSYGEDGPVFCGLNSDGSHLVACFGADASVLYGAPPNIPFLGLTAGDGFVCGLLLDTRQPYCWGSNSYVKSGVPQPMVEGARYSELSAGDNHLCALRAAQDGGRGSSAATSLIDCWGYNMTATHAVDEAVSTVSAGSVFNCGLFARNRTVFCWGDETVSGVVGLAPRDLHFQSIGAGGYHVCGVLENAQVFCWGRSLEMQQVVPSSAIGDGDVNIVPMDAMSTVVGGRFHACGIRSLDHQVACWGFTLHNSTSPPKGLKMYALVAGDYFTCGVPAETSLMPRCWGNSGPLALPMAVPPGICVPTACSHGYYEYVNHGEVGSIKVCKPANSRLCLPCSTGCPEGLYESSPCNATADRVCQFDCLKCVTDECLSFCLSQKRTKSRKLMAFQMRIFVAEIVFAVVLVLSVSVTTCLYVRHKLRHCQCSNRELRLAKSTAYSFRKDNMKIQPDMEDLKIRRAQEFSYEELEQATGGFSEDSQVGKGSFSCVFKGILRDGTVVAVKRAIKASDVKKSSKEFHNELDLLSRLNHAHLLNLLGYCEDGSERLLVYEFMAHGSLYQHLHGKDPNLKKRLNWARRVTIAVQAARGIEYLHGYACPPVIHRDIKSSNILIDEDHNARVADFGLSILGPADSGTPLSELPAGTLGYLDPEYYRLHYLTTKSDVYSFGVVLLEILSGRKAIDMQFEEGNIVEWAVPLIKAGDIFAILDPVLSPPSDLEALKKIASVACKCVRMRGKDRPSMDKVTTALEHALALLMGSPCIEQPILPTEVVLGSSRMHKVSQMSSNHSCSENELADGEDQGIGYRAPSWITFPSVTSSQRRKSSASEADIVGRRATDGRNVGSSIGDGLRSLEEEIAPASPQENLYLQHNF

>*Solanum lycopersicum* Solyc11g044940.1.1

MWKCRYFVRAFMFILIFSKVSGFGSMSSIAISYGEYGSVFCGLKSDGSHLVSCYGSTSSIIYSTPAHFPFIGLTAGNGFVCGLLMDSYQPYCWGKSNFVQMGVPQPMIKGSQYLEISAGENHLCGLRQPLMGKHRNTSLVDCWGYNMTTNNEFEGQIHSISAGSEFNCALFSVNKSVLCWGDETSSQVITLAPKDLRFIKIAAGGYHVCGILEGVNSQVYCWGRSMNLEEEFSVAQLNVELAAPSDPIISVVGGKFHACGIRSYDRHVVCWGYRVEKSTPPPSGVRFYEIAAGDYFSCGILAEISLLPVCWGFGFPSSLPLAVSPGVCKPRPCASGFYEFNNGTTTCKSPDSRICLPCTNGCPAEMYQQVECSSSRDSQCTYNCSSCTSVDCINNCSTAVSGKKNAKFWSLQLPVIVAEVAFAVFLVSVVSLTSIVYVRYKLRNCRCSGRSPSPRKNGSFPKEIAKDRADLDDLKIRRAQMFTYEDLERATEGFKEESQVGKGSFSCVFKGVLKDGTVVAVKRAIMSSDMKKNSKEFHTELDLLSRLNHAHLLNLLGYCEEGGERLLVYEYMANGSLHEHLHGKKKEQLDWIRRVTIAVQAARGIEYLHGYACPPVIHRDIKSSNILIDEEHNARVADFGLSLLGPANSSSPLAELPAGTLGYLDPEYYRLHYLTTKSDVYSFGVLLLEILSGRKAIDMQYDEGNIVEWAVPLIKAGDIEAILDPVLKPPSDAEALRRIANIASKCVRMRGKERPSMDKVTTALERALAQLMGSPSNDQPILPTEVVLGSSRMHKKSSSNRSTSETTDVAETEDQRYVEFRAPSWITFPSVASSQRRKSSVSDADVEAKNLESRNCGNGTDGLRSLEEEIGPASPHEHLFLKHNF

>*Brachypodium distachyon* KQK16646

MGSALALALCCVVFSLLLLPSWVDGLGSMGSISVSYGEDGPVFCGLSSDGSHLVACSGADATVVYAAPPRIRFLGLTAGDGFACGLLYDTSQPYCWGSNAYVQIGVPQPMLEGARYSEISAGDNHLCALRVPAKGADSSTIDCWGYNMTATHDVSGAVSTISAGSVFNCGLFARNRTVFCWGDETVSGVVELAPRNVRFQSIGAGGYHVCGVLENAQVFCWGRSLEMQQVSTTGAIGQGDVSIVPMDAMVSVVGGRFHACGIKSLDHQVACWGFTLQNSTAAPKGLRVYAIVAGDYFTCGVPAETSQKPRCWGHTGSSLLPMAVSPGLCVSVACSPGYYEYTSNGEVGGTKACKPANSRLCLPCSVGCPDGSYESSACNATADRVCQFDCSKCDSDECVSFCLSQKQTKNHKFIAFQMRIFVAEIAFAIILILTVTIIACLYVRHKLRHCRCSKSKLRMVKSTTYSFRKDNMKIQPDVEDLKIRTAQEFSYEELEQATGGFSEDSQVGKGSFSCVFKGILRDETVVAVKRAIKVSDVKKSSKDFHTELDLLSRLNHAHLLNLLGYCEDGSERLLVYEFMAHGSLYQHLHGKDPNLKKQLNWTRRVTIAVQAARGIEYLHGYACPPVIHRDIKSSNILIDEDHNARVADFGLSIMGPVDSGTPLSELPAGTLGYLDPEYYRLHYLTTKSDVYSFGVVLLEMLSGRKAIDMQCEEGNIVEWAVPLIKAGDISSILDPALSPPSDLEALKKIAAVACKCVRMRGKDRPSMDKVTTSLERALALLMGSPCMEQPILPTEVVLGSNRMHKKVSQRSSNQSCSENELIDGDDQRIEYRAPSWITFPSVTSSQRRKSSASEADLDGRTTTDGRNIGSSIGDGLRSLEEEIGPASPQEDLYLQHNF

>*Aegilops tauschii* AET7Gv20752400.1

MDSVPALALCCLILLPSWANGLGSMGSISVSYGEDGPVFCGLSSDGSHLVTCSGADASVVYGAPLRIPFLGLTAGDGFACGLLLDTSQPYCWGSNSYVKIGVPQPMVEGVEYSMLSAGDNHLCALRTPDKGIPRGVNPDTSVIDCWGYNMTATHVVAGAVSTISAGSVFNCALFARNRTVFCWGDETVSGVIGLAPRNVKFQSIGAGGYHVCGVLENAQVFCWGRSLEMQQVSPTGAIGEGDVSIVPMDAMVSVVGGRFHACGIRSLDHQVACWGFQLQNSTSAPKGLRLYTIVAGDYFTCGVPAETSMKPRCWGNSGPLALPMAVSPGICVSSACSPGYYEYVNHGELGSSKSCKPGNSRLCLPCSAGCPDNSYESSPCNATADRVCQFDCLRCVSDECLSYCTSRKQTNNHKSMDFQMRIFVAEIAFAVILIFTVTAISCLYVRHKLRDCRCSKSKLRMTKSTTYSFRKDNTKIQPDVEDLKIRRAQEFSYEELEQATDGFSEDSQVGKGSFSCVFRGILRDGTVVAVKRAIKVSDAKKSSKEFHTELDLLSRLNHAHLLDLLGYCEDGSERLLVYEFMAHGSLYQHLHGKDSNLKKQLNWTRRVTIAVQAARGIEYLHGYACPPVIHRDIKSSNILIDEDHNARVADFGLSIMGPVDSGTPLSELPAGTLGYLDPEYYRLHYLTTKSDVYSFGVVLLEILSGRKAIDMQLEEGNIVEWAAPLIKAGDISGILDPALSPPSDLEALKKIAAVACKCVRMRGKDRPSMDKVTTSLERALALLMGSPCLEQPILPTEVVLGSSRMHKKVSQRSSNQSCSENELVDGDDQRIEYRAPSWITFPSVTSSQRRKSSASEADLDGRTTTDGRNVGSSIGDGLRSLEEEIGPASPQEDLYLQHNF

>*Brassica napus* BnaC08g48960D-201

MTRNFVFDKQIHSLSAGSEFNCGLSSKDKSVFCWGDENSSQVISLIPKETKFQKIAAGGYHVCGILDGLNSRVLCWGKSLEFEEEISGNSTGSDKILDLPPKEPLSTVVGGKFYACGIKRYDHSAVCWGFFVNKSTTPPRGMGFYDLAAGNYFTCGVPTGNGMPPVCWGLGFPASIPLAVSPGLCREAPCPPGSHILGGPCKSPGSHICLPCSSTCPPDMYQKSECTERSDQICAYNCSTCVSPDCSSNCSSSTSLSGDGKPRGKFWSMQLPIATAEIGFALLLTAVVSISAVLYVRYRLRHCRCSESDARSSKDSAFTKDNGRPDLDKLQKRRRARVFTYEELEKAAEGFKEESIVGKGSFSCVYKGVLRDGTTVAVKKAIMSSDKQKNSNEFRTELDLLSRLNHAHLLSLLGYCEEGGERLLVYEFMAHGSLYNHLHGKNKALKEQLDWVKRVTIAVQAARGIEYLHGYACPPVIHRDIKSSNILIDEEHNARVADFGLSLLGPVDSGSPLAELPAGTLGYLDPEYYRLHYLTTKSDVYSFGVLLLEILSGRKAIDMHYEEGNIVEWAVPLIKAGDITSILDPVLKQPTEVEALRRIVSVACKCVRMRGKDRPSMDKVTTSLERALAQLMGNPSSEQPILPTEVVLGSSRMHKKSWRIGSENTEFRGGSWITFPSVTSSQRRKSSASEGDVAEEVEDEGRKQQEALRSLEEEIGPASPGQSLFLHHNF

>*Setaria italica* KQK94374

MDHVLVLAVCLLVLLPGWACGLGSMSSISVAYGEDGPVFCGISSDGSHIVTCFGDDASVLYGAPPNIPFLGLTAGDGFVCGLLLDTRQPYCWGSNSYVKSGVPQPMIEGAKYSELSAGDNHLCALQAAAAEIHGPNAATSLIDCWGYNMTATYVIHEAVSTISAGSVFNCGLFARNRTVFCWGDETVSGVVGLAPRDVRFQSIGAGGYHVCGVLENAQVFCWGRSLEMQQVAPASAIGDGDVNIVPIDAMVSVVGGRFHACGIRSIDHQVACWGFALRNSTSPPKGLKMYALVAGDYFTCGVPAETSLTPRCWGNSGPMALPMAVPPGICVPTACSHGYYEYDNHGEVGSSKICKPENSRLCLPCSAGCPEDSYESSPCNATADRVCQYDCSKCVTDECMSFCISQKRTKTRKLIAFQMRIFVAEIVFAIVLVLSVSVIACLYVQHKLRHCQCSNNELRMAKSTAYSFRKDNMRIQPDVEDLKIRRAQEFSYEELEQATGGFSEDSQVGKGSFSCVFKGILRDGAVVAVKRAIKASDMKKSSKEFHNELDLLSRLNHAHLLNLLGYCEDGSERLLVYEFMAHGSLYQHLHGKDPNSKKRLNWARRVTIAVQAARGIEYLHGYACPPVIHRDIKSSNILIDEDHNARVADFGLSILGPPDSGTPLSELPAGTLGYLDPEYYRLHYLTTKSDVYSFGVVLLEILSGRKAIDMQFEEGNIVEWAVPLIKAGDIFSILDPVLSPPSDLEALKKIASVACKCVRMRGKDRPSMDKVTTALEHALALLMGSPCVEQPILPTEVVLGSSRMHKVSQMSSNHSCSENELADGEDQRIEYRAPSWITFPSVTSSQRRKSSASEADIAGRTTTEGRNVGSSIGDGLRSLEEEIGPASPQEKLYLQHNF

>*Cucumis sativus* XP_004152097.1

MGLFLLVDLVVSFLKKMCGWRGGLFVELVVFADMCLLVSGLGSMSPLAVSYGEKGPVFCGLKSDGSHLVNCFGSNSAITYGTPSHFPFIGLTAGDGFVCGLLLDSNQPYCWGSSGYVQMGVPQPMIKGAQYLEISAGDYHLCGLRTPLTGRRRNMSFVDCWGYNMTRTFAFDGPIESISAGSEFNCGLFSLNRTVFCWGDETSSRVISLIPKDMRFQKIASGGYHVCGILEGANSRAFCWGRSLDIEEEISVAYSGEGNVELVPVDPLASVVGGKFHACGIKSSDRGVICWGFTVKPSTPPPDGIKVYDIAAGDYFTCGILAEKSLLPVCWGLGYPTSLPLAVSPGICKATPCPPGFYEISQDKARCKSPNFHVCMPCSSACPPDMYLKVECSLKSDRQCEYNCSTCFSSECLSNCSSMLSNGMMGRKNGKYWPVQQLPVLVAEIAFAVFLVAIVSLTAILYVRYKLRNCHCSGKELKSKKNKGTASSFQKESYKIRPDLDELKIRRAQMFTYEELERATCGFKEESIVGKGSFSCVFRGVLKDGTVVAVKRAIMSPNMQKNSKEFHTELDLLSRLNHAHLLNLLGYCEEGGERLLVYEFMAHGSLHQHLHGKNTALKEQLDWIRRVTIAVQAARGIEYLHGYACPPVIHRDIKSSNILIDEEHNARVADFGLSLLGPTDSSSPLAELPAGTLGYLDPEYYRLHYLTTKSDVYSFGVLLLEILSGRKAIDMQYEEGNIVEWAVPLIRSGDISAILDPILKPPSDAEALKRIANVACKCVRMRAKERPSMDKVTTALERALAQLMGSPCNEQPILPTEVVLGSSRLHKKSSQRSSNRSVSETDIAEAEDQRFEFRAPSWITFPSVTSSQRRKSSVSEADVDGKNLEGKNVGNCGGVGDGLKSLEEEIGPASPQEKLFLEHNF

>*Medicago truncatula* XP_003597519.1

MGFSRKQFSTYLLNTILFEVVVFSWLWSKVTGLGSMSSIAVSYGDKGSAFCGLKSDGSHTVTCYGMNSAIVYGTPSQFPFLGLTSGDGFVCGLLMSSNQPYCWGSSSHIEMGVPQPMFKDAQYLEISAGDYHVCGLRKPLTGRHRNFSFVDCWGYNMTKNYVFDGQIQSISAGSEFNCGLFSQNRTVFCWGNEVSTQVIRLIPQRMRFQKVSCGGYHVCGILEGVNSRTVCWGRSLGLEQEISLIPNQGQGGNVELAPNDPMLSVVGGKFHACGIKSYDHVVICWGLNLKTSTKVPKGIKVFDIAAGDYFTCGILAAKSLESICWGVGFPTSLPLAVSPRTRKCLSAPCPPSYYEIEKDQQNGLICQDPNSHLCVPCSGVCPDEMYQKSGCNLKSDILCEYNCSVCSSPECFSNCSSSSSNAANGGKKNERFWSMQLIVIVGEIVFAVFIVSAVSITAVMYVRYKLRDCECSTRPLNSMKRLNVSSSVQKDNGKVRPDAEEIKIRRAQKFSYEELENATCGFKEESIVGKGSFSCVFKGVLKDGTVVAVKRAIMSPNMQKNSKEFHTELDLLSRLNHAHLLNLLGYCEEGGERLLVYEYMAHGSLHQHLHGKNKELKEQLDWIRRVTIAVQAARGIEYLHGYACPPVIHRDIKSSNILIDEEHNARVSDFGLSLLGPTDSSSPLAELPAGTLGYLDPEYYRLHYLTTKSDVYSFGVLLLEILSGRKAIDMQYEEGNIVQWSVPLIKSGDIASILDPCLKPPSDIEALRRIANVACKCVRMRGKDRPSMDKVTTSLERALAMLMGSPCIDQPILPTEVVLGSNRMHKKTSQRSSNRSASEIDVVEGEDQRFEFRAPSWITFPSVTSSQRRKSSGSEGEVEVKIVEGRNYGNVVGGGGDVLRSLEEEIGPASPQERLFLQHNF

>*Solanum tuberosum* XP_006352076.1

MDVFNKWCVVMWKCRYFVRAFMLLVVFSKVSGFGSMSSIAISYGEYGSVFCGLKSDGSHLVSCYGSTSSIIYSTPAHFPFIGLTAGNGFVCGLLMDSYQPYCWGKSNFVQMGVPQPMIKGSQYLEISAGENHLCGLRQPLMGKHRNTSLVDCWGYNMTTNNEFEGQIHSISAGSEFNCALFSVNKSVLCWGDETSSQVITLAPKDLRFIKIAAGGYHVCGILEGVNSQVYCWGRSMNLEEEFSVAQLNVELAAPSDPIISVVGGKFHACGIRSYDRHVVCWGYRVEKSTPPPSGVRLYEIAAGDYFTCGILAEISLLPVCWGFGFPSSLPLAVSPGVCKPRPCASGFYEFNNGSATCKSPDSRICLPCTNGCPAEMYQQVQCTSSTDSQCTYNCSSCTSVDCLNSCSTAISGKKNAKFWSLQLPVIVAEVAFAVFLVSVVSLTSIVYVRYKLRNCRCSGKGPSPRKNGTFPKEIAKDRADLDDLKIRRAQMFTYEDLERATEGFKEESQVGKGSFSCVFKGVLKDGTVVAVKRAIMSSDMKKNSKEFHNELDLLSRLNHAHLLNLLGYCEEGGERLLVYEYMANGSLHEHLHGKKKEQLDWIRRVTIAVQAARGIEYLHGYACPPVIHRDIKSSNILIDEEHNARVADFGLSLLGPANSSSPLAELPAGTLGYLDPEYYRLHYLTTKSDVYSFGVLLLEILSGRKAIDMQYDEGNIVEWAVPLIKAGEIEAILDPVLKSPSDAEALRRIANIASKCVRMRGKERPSMDKVTTALERALAQLMGSPSNDQPILPTEVVLGSSRMHKKSSSNRSTSETTDVAETEDQRYVEFRAPSWITFPSVASSQRRKSSVSDADVEAKNLESRNCGNGTDGLRSLEEEIGPASPHEHLFLKHNF

>*Vitis vinifera* XP_002264936.1

MGISRSLLEHFLNWVLKIQTWQAVFLVQIRVLVVFSNLWWLVSGLGSMSSIAISYGENGPVFCGLKSDGSHLVTCYGSNSAIIYGTPAHFPFMGLTAGDGFVCGLLVDSNQPYCWGSSRYVQMGVPQPMIKGAEYLEISAGDYHLCGLREPLTGRLRNYSLVDCWGYNMTRSYRFDGQLQSISAGSEFNCGLFSQNRTVFCWGDETSSRVTSLIPQEMRFQKIAAGGYHVCGILEGANSRVFCWGGRSLDIEEEISTAYTGQGNVDSAPKDPMLSVVGGKFHACGIRSSDRGVTCWGFRVKTSTLPPDGIKVYEIAAGNYFTCGILAEKSLLPVCWGLGFPSSLPLAVSPGLCTPSPCLPGFYEFNHESPPCKSLNSHVCLPCSSACLDDMYQKAECTLKSDRQCEFNCSGCYSAECFSNCSSSSYANAITGRKTERFWSLQLPVVVAEVAFAVFLVSIVSLTTILYVRYKLRNCRCSDKGLKSKKGKANGSSFQNDNSKIRPDLDELKIRRAQTFTYDELERATGGFKEESQVGKGSFSCVFKGVLKDGTVVAVKRATMSSDMKKNSKEFHTELDLLSRLNHAHLLNLLGYCEEGGERLLVYEFMAHGSLHQHLHGKNKALKEQLDWVRRVTIAVQAARGIEYLHGYACPPVIHRDIKSSNILIDEEHNARVADFGLSLLGPADSGSPLAEPPAGTFGYLDPEYYRLHYLTTKSDVYSFGVLLLEILSGRKAIDMQFDEGNIVEWAVPLIKSGDISAILDPVLKPPSDLEALKRIATVAYKCVRMRGKERPSMDKVTTALERALAQLMGSPCNEQPILPTEVVLGSSRLHKKSSQRSSNRSVSETDVAEAEDQRFEFRAPSWITFPSVASSQRRKSSVSEADVDGKNLEARNLGSGGNGGDGLRSLEEEIGPASPQENLFLQHNF
